# Prevalence of osteoporosis in patients with anorexia nervosa: a systematic review

**DOI:** 10.1192/j.eurpsy.2026.11366

**Published:** 2026-07-31

**Authors:** I. Ramos-Suarez, I. Martin-Martin

**Affiliations:** 1 Psichiatry, Hospital Universitario San Cecilio; 2 Rheumatology, Hospital Universitario Virgen De Las Nieves, Granada, Spain

## Abstract

**Introduction:**

Anorexia nervosa (AN) is a severe psychiatric disorder characterized by restrictive eating behaviors, low body weight, and endocrine dysfunction, often resulting in significant medical complications. One of the most serious long-term consequences is osteoporosis, which affects bone mineral density and increases fracture risk, even in young patients. Despite its clinical relevance, the reported prevalence of osteoporosis in individuals with AN varies considerably between studies, depending on sample characteristics and diagnostic methods.

**Objectives:**

This systematic review aims to:

1. Estimate the prevalence of osteoporosis in individuals diagnosed with anorexia nervosa.
2. Explore differences in prevalence based on patient characteristics such as age, sex, and body mass index (BMI).

**Methods:**

A systematic search will be conducted in PubMed and Science Citation Index (SCI) to identify studies published in English or Spanish since January 1, 2010. Eligible studies must report original data on individuals with a clinical diagnosis of AN (according to DSM or ICD criteria) and provide osteoporosis prevalence data based on bone mineral density (BMD) measurements, typically using dual-energy X-ray absorptiometry (DXA) and a T-score ≤ –2.5. Two reviewers will independently screen and extract data, resolving disagreements through discussion. Risk of bias will be assessed using the Joanna Briggs Institute (JBI) Critical Appraisal Checklist for Prevalence Studies. PROSPERO LINK: https://www.crd.york.ac.uk/PROSPERO/view/CRD420251090224

**Results:**

The review is currently ongoing. Preliminary analysis of retrieved articles suggests notable variability in reported osteoporosis prevalence, likely influenced by differences in sample age, illness duration, and diagnostic approaches. Full quantitative results, including pooled prevalence estimates and subgroup analyses, will be presented upon completion of data synthesis.

**Conclusions:**

This will be the first systematic review focused specifically on the prevalence of osteoporosis in individuals with anorexia nervosa using recent evidence. The findings aim to inform clinicians, researchers, and policymakers about the magnitude of bone health impairment in this vulnerable population and to support the development of targeted prevention and monitoring strategies in psychiatric and medical care.

**Disclosure of Interest:**

None Declared

